# The Role of EGFR Amplification in Deep Venous Thrombosis Occurrence in IDH Wild-Type Glioblastoma

**DOI:** 10.3390/curroncol30050373

**Published:** 2023-05-12

**Authors:** Brandon Kaye, Assad Ali, Raphael Augusto Correa Bastianon Santiago, Bilal Ibrahim, Julio Isidor, Hany Awad, Mohammadmahdi Sabahi, Michal Obrzut, Badih Adada, Surabhi Ranjan, Hamid Borghei-Razavi

**Affiliations:** 1 Dr. Kiran C. Patel College of Allopathic Medicine, Nova Southeastern University, Fort Lauderdale, FL 33328, USA; bk694@mynsu.nova.edu; 2 Cleveland Clinic Florida, Department of Neurosurgery, Weston, FL 33331, USAsantiar6@ccf.org (R.A.C.B.S.); bilal.ibrahim@bau.edu.jo (B.I.); sabahim2@ccf.org (M.S.); ranjans@ccf.org (S.R.)

**Keywords:** epidermal growth factor receptor, venous thromboembolism, glioblastoma, IDH wild-type

## Abstract

**Introduction:** Glioblastoma (GBM) patients have a 20–30 incidence of venous thromboembolic events. EGFR is a widely used prognostic marker for many cancers. Recent lung cancer studies have described relationships between EGFR amplification and an increased incidence of thromboembolic complications. We aim to explore this relationship in glioblastoma patients. **Methods**: Two hundred ninety-three consecutive patients with IDH wild-type GBM were included in the analysis. The amplification status of EGFR was measured using fluorescence in situ hybridization (FISH). Centromere 7 (CEP7) expression was recorded to calculate the EGFR-to-CEP7 ratio. All data were collected retrospectively through chart review. Molecular data were obtained through the surgical pathology report at the time of biopsy. **Results:** There were 112 subjects who were EGFR-amplified (38.2%) and 181 who were non-amplified (61.8%). EGFR amplification status was not significantly correlated with VTE risk overall (*p* = 0.2001). There was no statistically significant association between VTE and EGFR status after controlling for Bevacizumab therapy (*p* = 0.1626). EGFR non-amplified status was associated with an increased VTE risk in subjects greater than 60 years of age (*p* = 0.048). **Conclusions***:* There was no significant difference in occurrence of VTE in patients with glioblastoma, regardless of EGFR amplification status. Patients older than 60 years of age with EGFR amplification experienced a lower rate of VTE, contrary to some reports on non-small-cell lung cancer linking EGFR amplification to VTE risk.

## 1. Introduction

Glioblastoma (GBM) has challenged neurosurgeons and neuro-oncologists worldwide due to a poor prognosis despite maximal safe resection, chemotherapy, or radiation [1]. The increased morbidity and mortality can be linked, in part, to the high incidence of venous thromboembolism (VTE) that occurs in roughly 20% of patients [2]. In certain populations, the incidence of VTE has been reported to be as high as 60% [3,4]. The association between cancer and VTE was established more than a century ago, but its complexity and variability across cancers has made it difficult to standardize a therapeutic management algorithm [5]. Tumor-associated thrombosis is a systemic condition with many potential influences, which explains the potential for distant emboli to form far from the primary lesion. The myriad factors that increase the risk of VTE in malignancy have been hypothesized to include TF produced by the tumor, which can activate the coagulation cascade, compression of vessels by the tumors with associated venous stasis, and prolonged immobilization of the patient, amongst others [6,7]. Cancer-associated thromboembolism presents a multitude of dangers to cancer patients and is a frequent cause of both increased morbidity and mortality [8,9]. As a result, researchers have been actively trying to understand the mechanisms of the malignancy-associated hypercoagulable state. Specifically concerning GBM, studies investigating the high rate of VTE are plentiful, yet the molecular, epigenetics, genomics data represent an area of active research. Research implicating specific mutations in GBM associated with higher incidence of VTE is scarce, making a consensus on patient management difficult. The pathophysiology of the hypercoagulable state has several possible etiologies, such as an imbalance between procoagulant factors and coagulant inhibitors as well as individual patient characteristics, including prolonged immobilization, obesity, smoking status, and the use of corticosteroids [3,10]. The high VTE incidence among these patients prompted Lim et al. to develop a score to predict the likelihood of developing symptomatic thromboembolism in patients with GBM on the basis of four variables: the Karnofsky Performance Scale (KPS), age, smoking, and hypertension [11]. This has opened the door for tailored treatment plans. However, even in the wake of advancements in diagnostic tools, therapeutic progress has been limited due to tumor resistance to new treatment paradigms [12].

Molecular analysis of cells has come to the forefront of modern oncological research and classifications. The cancer genome atlas (TCGA) program classified at least four subtypes of GBM on the basis of the main signature genes, including proneural, mesenchymal, neural, and classical. Each subtype has a unique variable response to treatment [13]. These biomolecular advancements have translated to further classification of GBM. Using molecular markers, Stupp et al. published the now highly regarded protocol for treating GBM on the basis of O6-methylguanine (O6-MeG)-DNA methyltransferase (MGMT) status [14]. This marker remains one of the strongest prognostic factors of survival for GBM and has been heralded as one of the most significant accomplishments in oncologic medicine for GBM [15]. While MGMT remains a key clinical marker for overall survival (OS), many other markers, such as Isocitrate dehydrogenase (IDH), epidermal growth factor receptor (EGFR), Alpha thalassemia/mental retardation syndrome X-linked (ATRX), phosphatase and tensin homolog (PTEN), are now commonly used in treatment planning [16].

EGFR promotes cell differentiation and proliferation, being expressed in the majority of human cells. During development, normal physiology, and in a multitude of pathological states, EGFR is indicated to be involved directly or in a peripheral role [17]. However, it was three decades ago when the discovery of EGFR mutations and overexpression in cancer cells increased its clinical value, opening the gate to therapies targeting it precisely [18]. EGFR is a widely used marker for the prognosis of many cancers [19,20,21,22]. Specifically, for GBM, EGFR amplification became the subject of a study in the classical subtype [23]. This culminated in the inclusion of EGFR amplification in the C-IMPACT-NOW Update 3 diagnostic criteria in 2018. IDH wild-type diffuse astrocytic glioma was then defined as containing EGFR amplification, telomerase promoter (TERTp) mutation, or gain of chromosome 7 and whole loss of chromosome 10 [24]. Of note, EGFR is present on chromosome 7. Only one of the aforementioned molecular changes is necessary to make the diagnosis per C-IMPACT-NOW. This change enabled histologically low-grade gliomas to be classified as more aggressive, high-grade gliomas, requiring more intensive treatments [25]. In addition to glioma classifications, EGFR has continued to be a topic of interest for clinical decision-making. More recent studies have been investigating EGFR amplification as an independent prognostic factor. Armacita et al. and Hoffman et al. both independently came to the conclusion that EGFR can play a role as an independent prognostic indicator in all age groups, regardless of other factors [26,27]. This makes EGFR a prime target for further investigation, as EGFR amplification status is frequently analyzed along with other prognostic factors for GBM patients. Given that these data are readily available, many are looking to expand the utility of patient EGFR status for patient treatment planning.

EGFR is known to enhance endothelial cell proliferation, including stimulating the production of growth factors, such as vascular endothelial growth factor (VEGF) [28]. VEGF can act as a chemotactic factor for cells that express tissue factor (TF), which may play a role in the coagulopathic state and lead to thrombosis, as mentioned above [29]. These effects may explain how therapies targeting EGFR can interfere with the coagulation cascade, producing deleterious effects in distant sites from the lesion. Recent studies on lung cancer have described an association between EGFR mutation and an increased incidence of DVT, PE, and other thromboembolic complications [30,31]. Additionally, recurrent GBM with EGFR amplification has been observed to demonstrate a worse response to bevacizumab, a known humanized monoclonal antibody that inhibits VEGF activity [32,33]. Therefore, significant cross-talk between EGFR and VEGF could be at the root of increased rates of VTE in GBM patients.

While links between EGFR alteration and thrombosis have been documented in other cancers, the role of EGFR in GBM VTE has yet to be described thoroughly. The aim of this study is to determine whether there is a relationship between EGFR amplification and VTE risk in GBM patients. Through retrospective analysis, we aim to evaluate the clinical outcomes and the incidence of DVT with amplification status of EGFR in GBM patients at a single surgical center to further understand the phenomenon of increased thrombotic risk.

## 2. Materials and Methods

This study is an IRB-approved retrospective analysis of the incidence of DVT, molecular markers, and clinical outcomes in a cohort of GBM patients at a single brain tumor center. Between 2015 and 2021, two-hundred and ninety-three consecutive patients with a confirmed diagnosis of IDH wild-type grade 4 glioma (GBM) were included in the study. Patients with IDH mutant status were excluded. All data were collected retrospectively through chart review. Molecular data were obtained through the surgical pathology report at the time of surgery. All patients underwent either unfractionated heparin or low-molecular-weight heparin (LMWH) treatment for one day following their neurosurgical procedures, per hospital protocol. Patient motor function was determined during neurological examination and was correlated to the lesion or surgical resection topology. The Karnofsky Performance Scale (KPS) scoring was used to determine patient functional status following surgical intervention. VTE events were recognized when patients were symptomatic, after which they were confirmed with ultrasound or computed tomography angiogram (CTA) for DVT and PE, respectively. EGFR amplification was determined by comparing expression with CEP7, after which the ratio EGFR/CEP7 was calculated. Univariate analyses were used to assess the differences in characteristics between EGFR non-amplified and EGFR-amplified groups, in which the Chi-square test or Fisher’s exact test were used to analyze the categorical variables, while the Wilcoxon rank sum test was conducted for continuous variables. Logistic regression analysis was performed to evaluate the difference in VTE event rate between the EGFR non-amplified and EGFR-amplified groups and for calculating OR (95% CI) of the significant variables. All data analyses were conducted using SAS version 9.4.

## 3. Results

Following analysis, 112 patients were categorized into the EGFR-amplified group, and 181 patients were in the EGFR non-amplified group. Binary logistic regression was performed to assess predictive value of different covariates for VTE occurrence overall in patients. After adjusting for covariates, both post-operative KPS score (0.96 OR, 95% CI 0.94–0.98; *p* = 0.0001) and Temozolomide treatment (0.214 OR, 95% CI 0.089–0.517; *p* = 0.001) were found to have statistically significant protective effects against the occurrence of VTE events. Conversely, pre-operative KPS was a significant predictor of VTE (1.022 OR, 95% CI 1.002–1.043; *p* = 0.032). Interestingly, the duration of hospitalization did not significantly predict the rate of VTE occurrence (1.005 OR, 95% CI 0.912–1.107; *p* = 0.918). Following this binary logistic regression analysis of VTE occurrence, no statistical significance was observed for the other covariates. Additional multivariate analysis was performed for the time of VTE occurrence relative to the surgical intervention (stereotactic biopsy with or without gross-total resection or subtotal resection). Results of this analysis showed that there was no statistically significant correlation between the VTE and the timing of the surgical intervention performed that was able to predict the occurrence of VTE events (Table 1). 

Subsequent analysis focused on the differences between EGFR-amplified and non-amplified EGFR subgroups. Univariate analyses (Table 2) showed that only one factor (bevacizumab adjuvant therapy) was significantly imbalanced between the EGFR non-amplified and EGFR-amplified groups. Multivariate logistic regression demonstrated that the difference in VTE events between EGFR non-amplified and EGFR-amplified groups was not significant (EGFR non-amplified vs. EGFR-amplified: OR = 1.37, *p* = 0.2001) when adjusted for the significant imbalance factor of bevacizumab (Table 3).

Further investigation revealed an association between EGFR amplification and patients above the age of 60 years. Univariate analysis was conducted for this age group and indicated that no factors were significantly imbalanced between the EGFR not-amplified and EGFR-amplified sub-groups (Table 4). Logistic regression analysis demonstrated that the difference in VTE events between the EGFR non-amplified and EGFR-amplified sub-groups for patients older than 60 years of age was significant, with EGFR non-amplified status patients being more likely to experience a VTE event compared with EGFR-amplified patients (OR = 1.83, *p* = 0.0480) (Table 5).

## 4. Discussion

GBM is the most common primary brain tumor and is notable for its highly aggressive nature [34]. More recent studies indicate that the incidence of GBM may be increasing, further highlighting the importance of proper management and treatment of patients living with these tumors [35,36]. Associated with GBM is a significant risk for thromboembolism, with upwards of 20–30% of patients experiencing a VTE each year [2,37]. Efforts to determine the biochemical mechanisms and pathways involved to reduce patient morbidity and mortality are underway. However, there are limited advancements in understanding of GBM and malignancy-associated thrombosis. As technology advances our understanding of malignancy using molecular and genetic analysis, we have begun to redefine the classification of cancers, as demonstrated by the 2021 WHO classification of gliomas [38]. These evolving technologies have allowed for a wealth of information that may shed light onto complex questions, including the relationship between VTE and GBM. 

Increasing scrutiny has been applied to EGFR and PTEN in the induction of tissue factor (TF) with malignancy-associated thrombosis, leading our team to determine whether any correlations existed in our patient population between EGFR and VTE. EGFR is often thought of as an angiogenic and metastatic regulator for cancers, allowing more perfusion with blood to encourage proliferation and spread to distant sites [39]. EGFR induction of TF, a prothrombotic receptor and cofactor for factor VII/VIIa in the coagulation cascade, has also been hypothesized to increase thrombosis with malignancies. Increased thrombosis with EGFR alterations has been observed in GBM and NSCLC, supporting this hypothesis [30,40,41,42,43]. It is important to note that the alterations in GBM and NSCLC are fundamentally different. As Lin et al. detailed in their analysis, the mutations that have been recognized in NSCLC alter the tyrosine kinase domain [44]. Determining this mutational status in random biopsy is sufficient to initiate EGFR-targeted treatments. GBM, on the other hand, has considerable heterogeneity, with multiple different EGFR variants affecting the extracellular domain of the receptor [44]. There are no known gatekeeping functions that this receptor performs for GBM, even with these mutations. This complicates the use of EGFR as a treatment target, as the response of tumors to anti-EGFR treatments cannot be readily determined by a single biopsy, as seen in NSCLC. The pathways involved in treatment have yet to be elucidated, making the role of EGFR in GBM unclear concerning both the survival of tumor cells and the potential for thromboembolic complications. 

The role of EGFR in areas other than targeted drug therapies is still expanding. EGFR is often recorded in pathology reports due to its increasing use as a prognostic indicator, highlighting its importance in the progression of cancer. We believed that if there was an association between EGFR and VTE, it could be clinically useful because these data are often readily available. Therefore, we hypothesized that EGFR alteration could prognosticate VTE risk in glioblastoma patients on the basis of some findings in NSCLC. This research used patient data in this manner to look for trends that could be utilized in the clinical setting. However, univariate analysis results indicated no correlation overall between EGFR amplification and higher incidence of VTE, as reported in other studies (Table 3). Though our finding is contrary to what is found in most studies on EGFR-mutated lung cancer, it is similar to a study on 310 lung cancer patients, which noted a protective effect of EGFR-mutated patients on DVT risk [45]. In addition, these findings are in line with other authors who have indicated that EGFR is not significantly associated with EGFR amplification status or, at best, plays a minor role compared with other mutations [45,46,47,48]. Determining the cause of this inconsistency may require careful analysis of the study designs and the variables at play in EGFR research. Highlighting this, Alexander and Burbury determined that studies describing an increased risk of VTE with EGFR expression often do not provide a timeline for their thrombotic complications relative to the cancer diagnosis and treatment [49]. They also stated that EGFR-targeted treatments themselves may introduce variables that affect study results [49]. Careful analysis of individual patient characteristics and the multitude of variables associated with cancer treatment may establish a more concrete explanation of the role of EGFR and VTE.

Initial analysis of the risk factors and demographics was necessary to investigate potential trends between VTE and this population of patients. Our analysis encompassed many covariates in the data, but only a few trends were significant in our linear regression analysis. These primarily included medication exposure and patient functional status. Firstly, adjuvant temozolomide therapy was indicated to play a protective role in preventing DVT in these patients (Table 1). This finding is contradictory to other works in the literature, whereby the treatment of high-grade glioma with temozolomide was associated with increased rates of VTE [50,51]. In vitro studies have also linked TF release from treated GBM cells to this increase in thrombosis [52]. Although our results may be due to reduced tumor burden and, therefore, reduced incidence of prothrombotic mediators, further investigation may help to explain these contradictory results. Concerning patient function, we opted to use the Karnofsky Performance Scale as a quantitative measure of dysfunction. As patients who are neurologically compromised may have reduced mobility, this could contribute to venous stasis. Other authors have noted that reduced KPS and motor function in glioma and other intracranial lesions increase the risk of VTE and DVT [53,54]. Our analysis indicated that in both the pre-operative and post-operative periods, there were significant correlations between thromboembolic events and KPS values. For example, increased pre-operative KPS was associated with a lower incidence of VTE, and higher post-operative KPS was associated with a protective effect against VTE (Table 1). We believe that the systemic effects of TF released from GBM or tumor-derived secondary messengers may be compounded by the patient functional status and associated venous stasis. Interestingly, although KPS was noted to play a role in VTE occurrence, the total length of hospital stay was not associated with an individual’s VTE risk (Table 1). Our data did not directly explain why this may be the case; however, patient functional status was not necessarily directly tied to the patient’s length of stay. It may be that the multitude of factors that influenced the need for a longer admission, combined with active measures to mitigate DVT in hospitalized patients, influenced the occurrence of VTE in the perioperative period [55]. Consistent with these results, many patients experienced VTE months after their procedures, with only 28 patients experiencing an event preoperatively or during the inpatient, perioperative period. Due to the factors mentioned previously with malignancy, we attribute these effects most likely to be the result of a systemic hypercoagulable state induced by GBM. This state, combined with reduced KPS, may explain why many patients experienced their VTE events months or years after their procedures. No other demographic, risk factors, or comorbidities were recognized as having a significant association in the binary or multivariate regression analyses.

Further multivariate analysis was utilized to determine whether there were any confounding variables in our EGFR-amplified and non-amplified patient populations specifically. There was a significant difference in the patient groups taking bevacizumab therapy, with 42.0% EGFR-amplified patients taking the medication, compared with 28.7% of the EGFR non-amplified group. Bevacizumab is a humanized monoclonal antibody against VEGF, and VEGF has been hypothesized to have a similar function to EGFR in angiogenesis and thrombosis. Therefore, any imbalance in anti-VEGF treatment needed to be addressed in this patient population. Furthermore, EGFR and VEGF have been implicated in similar angiogenic and thrombotic signaling cascades during malignancy, some of which are redundant [29,56,57,58,59]. EGFR is thought to activate transcription factors that stimulate the production of VEGF, leading to an autocrine activation of the VEGFR and increased angiogenesis [56,60]. As VEGF has been implicated in the same signaling cascades with TF as EGFR, we sought to determine whether bevacizumab therapy would influence our results. Bevacizumab adjuvant treatment in particular has been linked with increased thrombotic risk [33,61,62]. Multivariate analysis demonstrated there was not a statistically significant difference in our results when controlling for bevacizumab therapy (Table 3). Although others may have observed correlations in their data between bevacizumab treatment and thrombotic risk, we did not observe any notable difference between the EGFR-amplified and non-amplified groups.

Although our results indicated no influence between EGFR alteration and VTE overall, there was a trend in the data pertaining to patient age. EGFR non-amplified (wild-type) status was associated with an increased risk of thrombosis in patients >60 years old for this group of participants (Table 5). This implies that in patients >60 years, EGFR amplification may have had a protective effect on the risk of DVT. These results are similar to those published by Davidsson et al., whereby they determined EGFR may have played a protective role in VTE occurrence [45]. The causal association between EGFR alteration and VTE cannot be ascertained from these data because this is a retrospective review; however, these results raise an interesting question as to the role EGFR plays in VTE in older patients. Physiological responses and tumor pathophysiology varies with age for GBM patients. VTE risk increases with age, and older patients often present with higher grade tumors, another factor which also puts patients at risk of VTE [2,4,37,63,64,65]. One study by Dou et al. investigated EGFR mutation status and demonstrated an increased VTE risk in EGFR wild-type adenocarcinoma patients [66]. Other studies investigating anti-EGFR treatments have also noted an increased rate of thrombosis [33,61,62,67,68,69,70]. The split consensus for the role of EGFR in NSCLC-associated VTE described above may explain why studies that focused on EGFR in other malignancies have not shown conclusive results. Therefore, we believe that further research is necessary to understand the nuance of EGFR involvement in the prothrombotic state associated with GBM and other malignancies. As age was indicated to play a factor, further study into how prothrombotic pathways change with age will enable a better interpretation of our results. Stratification of patients on the basis of age may also allow for more personalized treatment and research to mitigate thrombosis risk in GBM patients.

Limitations of this study include the retrospective design, the number of participants, and the limited data set from only patients at a single institution. Having a greater number and variety of patients may have allowed for more nuanced trends to be recognized in the data. The method we used for detecting VTE may have influenced these results. Patients were recorded as having a thromboembolic event only on the basis of symptomatic VTE. Therefore, it is possible that VTE events may have been missed, potentially skewing the results. Because the study was retrospective, we were unable to determine whether these subclinical events occurred. Furthermore, we were unable to determine the cause for the trends we noted in the population of patients >60 years of age, as the study did not investigate the biochemical pathways involved. Although these data did not contain any trends which enable actionable prognostic or adjuvant therapy recommendations, further investigation may shed light onto other pathways related to thrombosis in GBM. 

## 5. Conclusions

Thrombosis in GBM presents an important clinical risk for patients, and the molecular signaling cascades that may influence this risk are still under investigation. Although EGFR is involved in angiogenesis and thrombosis, no significant risk factor has been determined between EGFR and VTE, except in patients older than 60 years of age, where we found that EGFR amplification had a protective effect on DVT risk. The hypothesized positive association between EGFR alteration and VTE was not consistent with the previously reported lung cancer literature, as EGFR non-amplified elderly glioblastoma patients were at an increased risk of VTE. Further prospective studies can investigate risk stratification by age group for different molecular markers of GBM.

## Figures and Tables

**Table 1:** Binary analysis of statistically significant covariates for VTE.

Variable	Odds Ratio	95% Confidence Interval	*p*-Value
Pre-operative KPS	1.022	1.002–1.043	0.032 *
Post-operative KPS	0.96	0.94–0.98	0.0001 *
Temozolomide Adjuvant Therapy	0.214	0.089–0.517	0.001 *
Length of Hospital Stay	1.005	0.912–1.107	0.918 *

* Statistically significant results.

**Table 2:** Univariate analysis for comparing the characteristic factors and VTE between the EGFR non-amplified and EGFR-amplified groups.

Variable	EGFR Non-Amplified	EGFR-Amplified	*p*-Value
	(*n* = 181)	(*n* = 112)	
**Age, median (range)**	64 (17–95)	64(35–84)	0.7376
Age, *n* (%)			0.7491
Age ≤ 60	68 (37.6)	40 (35.7)	
Age > 60	113 (62.4)	72 (64.3)	
Sex (male), *n* (%)	108 (59.7)	72 (64.3)	0.4301
Race, *n* (%)			0.8979
White	169 (93.4)	105 (93.7)	
Other	12 (6.6)	7 (6.3)	
BMI, median (range)	28.3 (16.7–61.6)	27.7 (17.6–48.1)	0.6885
Smoking, *n* (%)			0.3527
Never	97 (53.6)	67 (59.8)	
Current	13 (7.2)	8 (7.1)	
Former	67 (37.0)	37 (33.0)	
Not reported	4 (2.2)	0 (0.0)	
Connective tissue, *n* (%)	3 (1.7)	1 (0.9)	1
MI, *n* (%)	3 (1.7)	3 (2.7)	0.6779
CHF, *n* (%)	8 (4.4)	2 (1.8)	0.3272
PVD, *n* (%)	1 (0.6)	2 (1.8)	0.5603
COPD, *n* (%)	11 (6.1)	6 (5.4)	0.7978
Cerebrovascular disease, *n* (%)	75 (41.4)	54 (48.2)	0.2561
Leukemia, *n* (%)	1 (0.6)	0 (0.0)	1
Liver disease, *n* (%)	6 (3.3)	0 (0.0)	0.0858
Motor deficits before VTE, *n* (%)	19 (10.5)	15 (13.4)	0.452
Radiation therapy, *n* (%)	152 (84.0)	89 (79.5)	0.3258
Temozolomide, *n* (%)	141 (77.9)	92 (82.1)	0.3819
Bevacizumab, *n* (%)	52 (28.7)	47 (42.0)	0.0199 *
Steroid, *n* (%)	178 (98.3)	111 (99.1)	1
Outcome (VTE), *n* (%)	96 (53.0)	52 (46.4)	0.2715

* Statistically significant results.

**Table 3:** Multivariate Logistic Regression Analysis for VTE event.

Effect	Odds Ratio	95% CI	*p*-Value
EGFR (not-amplified vs. amplified)	1.37	(0.85–2.21)	0.2001
Bevacizumab (No vs. Yes)	0.7	(0.43–1.15)	0.1626

**Table 4:** Univariate analysis for comparing the characteristic factors and VTE between the EGFR non-amplified and EGFR-amplified sub-groups (age > 60).

Variable	EGFR Non-Amplified	EGFR-Amplified	*p*-Value
	(*n* = 113)	(*n* = 72)	
**Age, median (range)**	71 (61–95)	69 (61–84)	0.1947
Sex (male), *n* (%)	62 (54.9)	42 (58.3)	0.6431
Race, *n* (%)			0.4859
White	106 (93.8)	70 (97.2)	
Other	7 (6.2)	2 (2.8)	
BMI, median (range)	27.8 (16.7–61.6)	27.3 (17.7–48.1)	0.5176
Smoking, *n* (%)			0.193
Never	52 (46.0)	42 (58.3)	
Current	6 (5.3)	4 (5.6)	
Former	51 (45.1)	26 (36.1)	
Not reported	4 (3.5)	0 (0.0)	
Connective tissue, *n* (%)	2 (1.8)	1 (1.4)	1
MI, *n* (%)	3 (2.7)	3 (4.2)	0.6792
CHF, *n* (%)	6 (5.3)	2 (2.8)	0.4863
PVD, *n* (%)	1 (0.9)	2 (2.8)	0.5612
COPD, *n* (%)	11 (6.1)	6 (5.4)	0.7978
Cerebrovascular disease, *n* (%)	51 (45.1)	41 (56.9)	0.1172
Leukemia, *n* (%)	0 (0.0)	0 (0.0)	NA
Liver disease, *n* (%)	4 (3.5)	0 (0.0)	0.1581
Motor deficits before VTE, *n* (%)	11 (9.7)	9 (12.5)	0.5548
Radiation therapy, *n* (%)	90 (79.7)	56 (77.8)	0.7613
Temozolomide, *n* (%)	80 (70.8)	57 (79.2)	0.2054
Bevacizumab, *n* (%)	26 (23.0)	21 (29.2)	0.3482
Steroid, *n* (%)	110 (97.4)	72 (100.0)	0.283
Outcome (VTE), *n* (%)	64 (56.6)	30 (41.7)	0.0471

**Table 5:** Logistic Regression Analysis for VTE event for sub-groups (age > 60).

Effect	Odds Ratio	95% CI	*p*-Value
EGFR (not-amplified vs. amplified)	1.83	(1.01–3.33)	0.0480 *

* Statistically significant results.

## Data Availability

The data presented in this study are available upon request from the corresponding author. The study’s data collection form contains patient identifiers.
